# Association between continuity of care and attendance of post-discharge follow-up after psychiatric emergency presentation

**DOI:** 10.1038/s44184-023-00052-9

**Published:** 2024-02-06

**Authors:** Ben Hoi-Ching Wong, Petrina Chu, Paul Calaminus, Cathy Lavelle, Rafik Refaat, Dennis Ougrin

**Affiliations:** 1 East London NHS Foundation Trust, London, UK; 2 Queen Mary University of London, London, UK; 3 Institute of Psychiatry, Psychology & Neuroscience, King’s College London, London, UK

**Keywords:** Health policy, Health services

## Abstract

The number of accident and emergency (A&E) hospital attendances by young people aged 18 or under with a recorded diagnosis of a psychiatric condition more than tripled between 2010 and 2022. After discharge from the hospital, attendance at follow-up appointments in the community is critical to ensure the safety of young people and optimise the use of clinical resources. A retrospective cohort study was conducted to evaluate the association between follow-up attendance and the continuity of clinicians and clinical teams, using electronic clinical record data from East London NHS Foundation Trust (ELFT), between April 2019 and March 2022. Multi-level mixed effects logistic regression was performed to model the follow-up attendance odds based on whether the same or different clinician and clinical team offered the initial A&E and the community follow-up appointment or whether a crisis team was involved. 3134 A&E presentations by 2368 young people were identified within the study period. Following these presentations, 2091 follow-up appointments in the community were offered. The attendance rate increased by more than three times if the follow-up appointment was offered by the same clinician who saw the young person in A&E (odds ratio (OR) = 3.66; 95% CI 1.65–8.13). Whether the same clinical team provided the community follow-up appointment, or whether a crisis team was involved before discharge made no difference to the likelihood of follow-up attendance. The findings support the importance of the continuity of clinicians in the care of young people in crisis.

## Introduction

Non-attendance of out-patient appointments poses a significant burden to health services worldwide. A missed appointment without prior notice is commonly known as ‘did not attend’ (DNA). The latest data reported 7.8 million DNAs in England in 2021–22, costing the NHS up to £1 billion per year^1^. DNA rate in psychiatry is particularly high, estimated to be as high as 20%, and double that of other medical specialties^2–5^. This presents additional challenges from the clinical perspective. Patients who missed mental health appointments were found to be more unwell^6^, more likely to drop out of mental health service^7^, to have a higher likelihood of readmission to hospital^8^, and to exacerbate treatment waiting time for other patients^9^.

In children and adolescents, mental health conditions are a major cause of morbidity^10^ and are increasingly prevalent^11^. Since the COVID-19 pandemic, a global increase has been reported in young people presenting at accident and emergency departments (A&E) for self-harm and other psychiatric emergencies^12^. Post-discharge mental health follow-up for these young people is crucial. Previous research corroborated the elevated risks of completed suicide, hospital readmissions, and preventable adverse events, within the first week following hospital discharge^13–15^. Current NICE guidelines recommend follow-up within 7 days of discharge from inpatient settings^16^. However, follow-up attendance rates are low for those who present at A&E for self-harm^17^. In particular, low mental health service utilisation was observed in children from certain minority ethnic groups^18,19^ or deprived neighbourhoods^20^. In England, children and adolescent mental health services (CAMHS) are often reported to be underresourced and do not always have the capacity to effectively engage non-attenders^21^. Up to half of the young people who present at emergency units for psychiatric concerns have no previous mental health service contact^22^. Therefore, missed follow-up appointments likely imply missed opportunities to access any long-term mental health support.

The important question that follows is how post-discharge follow-up appointment attendance can be increased. Aside from the intuitive and effective answer of providing reminders^23,24^, factors at the service delivery level such as communication and therapeutic alliance have significant impacts on patient’s motivation to engage with mental health services^4,20,25^. Young people are more likely to attend subsequent mental health appointments when there is a shorter delay between the referral and the appointment^26^ if they are satisfied with the initial care^27^, the clinician has explained clearly the need for the appointment^28^ and persists in maintaining contact^29^. The first interaction between the patient and the mental health service is critical for establishing rapport for long-term treatment adherence^30^. Therapeutic interventions incorporating early engagement were demonstrated to promote follow-up attendance of young people who present with self-harm^31,32^. Care initiated as soon as possible at A&E with planned continuity into the community settings is both a national and international policy focus^33,34^. Mental health crisis response services have been developed to achieve this^35^. These teams commonly start engaging young people in hospitals, as soon as they present at A&E, and support planning for discharge.

To date, few high-quality studies have evaluated or compared the effectiveness of crisis services for children and adolescents^36^. Previous studies focused on clinical outcomes such as duration in hospital, depressive symptoms, and rate of rehospitalisation^37,38^. However, follow-up attendance, an equally important metric, had not been formally evaluated. Adult patients were found to be more likely to attend the first post-discharge appointment if they were seen at A&E by a community outreach mental health team^39^. Another essential yet unanswered research question lies within the operational aspect of follow-up care, which varies across localities and cases. In practice, whether the crisis team or another mental health team provides the follow-up appointment may depend on the service model, the psychosocial complexity, and the existing professionals around the young person. Whether the follow-up clinician has previously seen the patient at the hospital may depend on team capacity and rota. One may argue these uncertainties should be avoided, considering the benefits of continuity of care including reduced communication barriers^40^. Children and young people may be more inclined to attend follow-up appointments when they see the same mental health team and/or clinician across the hospital and the community.

The current study aimed to examine the post-discharge follow-up attendance of young people who presented with mental health crises in East London. We evaluated the association between their attendance and the involvement of CAMHS crisis services, and the effects of seeing the same clinician or same team at A&E and at follow-up.

## Methods

### Study design, setting and data sources

This is a retrospective cohort study, as part of service evaluation under the IVY trial (ISRCTN42999542). IVY is a randomised controlled trial that seeks to evaluate the clinical effectiveness and cost-effectiveness of intensive community care services. Crisis teams of the present study site, East London NHS Foundation Trust (ELFT), were included.

Routine clinical record data were collected using the ELFT electronic clinical system, *Rio*. Eligible A&E presentations were made (1) at any of the five hospitals within ELFT; (2) between 1 April 2019 and 31 March 2022; (3) by young people under 18 years old; and (4) for any psychiatric difficulties. All hospital contacts with CAMHS clinicians in the same spell for each eligible presentation were identified. Follow-up appointment was defined as the first scheduled, CAMHS outpatient, direct clinical contact, within 14 days of the last hospital contact following each eligible A&E presentation.

### CAMHS crisis services

The present study included five CAMHS crisis teams in ELFT (Bedfordshire/City and Hackney/Luton/Tower Hamlets/Newham), serving an estimated child population of 404,000^41^. Crisis teams are commonly multi-disciplinary, consisting of professionals such as mental health nurses, social workers, and psychiatrists. When indicated, crisis teams offer immediate psychiatric support for young people who present with any mental health crisis. Timely initial, risk and/or psychosocial assessments are conducted at A&E. Crisis teams may also support liaison between relevant mental health or social care professionals, identify appropriate home-based or community care, and assist in the formulation of a discharge plan.

### Outcome and comparisons

The following sociodemographic data were collected: Age, Ethnicity (white British/other), looked-after child status (yes/no), and home postcode. Deprivation decile according to the national statistics was retrieved using the postcode information^42^.

The primary outcome was follow-up appointment attendance, coded as one of the following: Attended, did not attend (DNA), cancelled by the client, or cancelled by the provider.

Binary variables were created, respectively, to identify the comparisons: (1) whether the follow-up appointment was offered by any crisis team, (2) whether it was offered by a clinical team that saw the patient at the hospital, (3) whether it was offered by a clinician that saw the patient at the hospital, and (4) whether the follow-up appointment followed an A&E presentation that involved self-harm. The borough of the clinical team was coded. It was also coded whether the follow-up appointment was scheduled to be a face-to-face contact or any other remote medium.

### Statistical methods

The socio-demographic characteristics of the young people who presented at A&E were summarised using descriptive statistics. The available data of the follow-up appointments resulting from these presentations were tabulated.

Predictors of missingness were assessed using logistic regression (Supplementary Tables 1, 2). Each variable was fitted to a summary variable indicating whether the record was complete. Predictors of missing follow-up appointment attendance data were also assessed (Supplementary Tables 3, 4).

To compare the attendance of follow-up appointments offered by the crisis teams with those offered by alternative CAMHS teams, multi-level mixed-effects logistic regression was adopted on the attendance outcome. Non-attendance was defined as DNA, whilst cancellations by the client or the provider were treated as missing data. Random intercepts were included for anonymised patient identifiers and boroughs of the A&E, with patients clustered within boroughs. This model was chosen to account for the similarities within individuals and CAMHS locations. Ethnicity, looked-after-child status, and deprivation decile were adjusted for since they are likely confounders for attendance rate as suggested by previous publications. Complete-case approach was adopted. The odds ratio (OR) and 95% confidence intervals (CI) were presented. The same model was repeated for the other three comparisons outlined above.

Sensitivity analyses were conducted to test the robustness of including only DNA as non-attendance. Routine errors (e.g. clinicians misidentifying DNAs as cancellations by patients) are common in data recording^43^. In practice, cancellations are not always requested with sufficient notice and clinical resources may not be redistributed^44^. The same regression models for the four variables of interest were repeated, with non-attendance defined as DNA or cancellation by patients. Cancellations by providers were excluded from the analysis.

### Ethical approval

This service evaluation was reviewed and approved by ELFT’s Governance and Ethics Committee for Studies and Evaluations (Ref.: G2208a). The utilised data were sufficiently anonymous and therefore participant consent was not applicable.

## Results

### Patient characteristics

Overall, 2368 young people, aged between 4 and 18 years, presented at the A&E in ELFT for psychiatric difficulties between 1 April 2019 and 31 March 2022. Table 1 summarises the demographic characteristics of these young people at referral. Over half (62%) of the patients at referral reported ‘Other ethnicity’. Four per cent of the sample were looked-after-children. Altogether, 72% of the young people came from neighbourhoods amongst the most deprived half (i.e. deciles 1–5) of the UK. Almost a fifth (19%) of the sample presented at A&E for psychiatric difficulties more than once during the study period.

**Table 1:** Summary of demographic characteristics of young people at referral.

Characteristic	Summary (*N* = 2368)
Age, median (quartiles), yr	15 (14–16)
*Ethnicity*	
White British	893 (38%)
Other	1473 (62%)
Not known	2 (0.08%)
*Self-harm at presentation*	
Yes	967 (41%)
No	366 (15%)
Not known	1035 (44%)
*Looked-after-child*	
Yes	105 (4%)
No	2262 (96%)
Not known	1 (0.04%)
*Deprivation decile*	
1	226 (10%)
2	497 (21%)
3	417 (18%)
4	327 (14%)
5	206 (9%)
6	170 (7%)
7	123 (5%)
8	129 (5%)
9	140 (6%)
10	114 (5%)
Not known	19 (0.8%)
*Repeated A&E presentations*	
Yes	450 (19%)
No	1918 (81%)

### Follow-up appointment characteristics

Out of a total of 3134 A&E presentations, post-discharge follow-up was recorded for 2091 (67%) presentations. Table 2 summarises the data of the follow-up appointments. Information on appointment attendance was available for 2023 appointments, of which 1761 (87%) were attended. Information on the teams that scheduled the first community follow-up was available for 2028 appointments, half of which (53%) were offered by crisis teams. In all but one instance, appointments that were attended had no missing data on appointment medium or team.

**Table 2:** Summary of follow-up appointment details.

Follow-up appointment detail (*n*)	Summary
*Attendance* (*n* = 2023)	
Attended	1761 (87%)
Did not attend (DNA)	139 (6.9%)
Cancelled by client	32 (1.6%)
Cancelled by provider/admitted to hospital	91 (4.5%)
*Crisis team at first follow-up* (*n* = 2028)	
Yes	1074 (53%)
No	954 (47%)
*Same clinical team as seen in hospital* (*n* = 2028)	
Yes	1051 (52%)
No	977 (48%)
*Same clinician as seen in hospital* (*n* = 2028)	
Yes	347 (17%)
No	1681 (83%)
*Appointment medium* (*n* = 1760)	
Face-to-face	1193 (68%)
Other	567 (32%)

### Attendance of follow-up appointments

Table 3 summarises the results of multi-level logistic regression analyses on the offered follow-up appointments following recorded A&E presentations. We observed no significant difference in attendance odds between follow-up appointments offered by crisis teams and those offered by other teams. Attendance odds were also not found to be associated with whether the clinical team at follow-up had previously seen the client at the hospital. However, there was a threefold increase in attendance of follow-up when it was offered by a clinician who had had direct contact with the client at the hospital (OR, 3.66; 95% CI, 1.65–8.13). A&E presentations that involved self-harm were not observed to result in any difference in follow-up attendance.

**Table 3:** Formal analyses of follow-up appointment attendance (non-attendance = DNA).

Variable	Adjusted OR for attendance
Appointment with a crisis team (*n* = 1879)	1.33 [0.85–2.07]
Same clinical team (*n* = 1879)	1.26 [0.81–1.95]
Same clinician (*n* = 1879)	3.66 [1.65–8.13]
Self-harm presentations (*n* = 934)	0.78 [0.40–1.50]

Cancellations by the patient or the provider were treated as missing entries; *n* = number of available observations after listwise deletion.

The sensitivity analyses utilised the same regression models, testing under the assumption that cancellations by patients were misidentified DNA in the clinical record or not requested with sufficient notice. Crisis teams were found to be associated with a 77% increase in attendance odds in the follow-up appointments they scheduled compared with other CAMHS services (OR, 1.77; 95% CI, 1.09–2.85). There was an observed benefit of improved attendance when the follow-up appointment was offered by the same clinical team (OR, 1.65; 95% CI, 1.03–2.64) or the same clinician (OR, 4.63; 95% CI, 1.98–10.81) who had seen the client at the hospital. There was no evidence of any significant difference in follow-up attendance odds from initial self-harm A&E presentation compared with non-self-harm presentations (Table 4).

**Table 4:** Sensitivity analyses of follow-up appointment attendance (non-attendance = DNA, or cancellations by the patient).

Variable	Adjusted OR for attendance
Appointment with a crisis team (*n* = 1911)	1.77 [1.09–2.85]
Same clinical team (*n* = 1911)	1.65 [1.03–2.64]
Same clinician (*n* = 1911)	4.63 [1.98–10.81]
Self-harm presentations (*n* = 957)	0.87 [0.46–1.66]

Cancellations by the provider were treated as missing entries; *n* = number of available observations after listwise deletion.

## Discussion

The present study demonstrates an important benefit of clinician continuity in the follow-up care of young people who present with mental health crises. Young people were at least three times more likely to attend the follow-up appointment in the community if they had already seen the designated clinician at the hospital before discharge. The attendance odds were potentially also increased by seeing the same clinical team, or the involvement of any crisis team, although results were not conclusive and only significant when cancellations by patients were taken into account. Self-harm was not found to be associated with any difference in follow-up attendance.

The current study is, to the best of our knowledge, the first to formally evaluate the associations between the CAMHS clinician or team identity at the time of psychiatric crisis presentation, and the attendance of the first post-discharge follow-up appointment. In previous studies, follow-up appointment attendance or engagement was targeted by bespoke brief interventions, such as reminder systems^23,24^, and facilitated service linkage using an external engagement agency^45^. The present findings suggest that changes can be made within the existing emergency mental health service model to achieve the same benefit. Prioritising the allocation of the same clinician across both A&E and community settings can likely boost the patient’s motivation to attend the first follow-up appointment in the community. Whilst this aligns with similar findings in adults^39^, relevant research in children is limited. Continuity of care was often studied as an outcome of appointment attendance rather than a factor^46^. Moreover, we observed a more obvious effect of continuity of care at the clinician level, compared with that at the team level. This conflicts with previous evidence, where mental health service dropouts were better accounted for by heterogeneity at the service level rather than at the practitioner level^47^. Despite previous suggestions of poor compliance with follow-up care among adolescents who self-harm^48^, we did not observe their attendance odds to be different compared with other adolescents.

The present findings are perhaps not entirely surprising to some practitioners. The importance of clinician continuity is routinely emphasised by young people in service evaluation^49^. It can be anxiety-provoking or tiring to re-invest emotionally and develop therapeutic relationships with multiple therapists. However, this preference is not always compatible with the design of service provision or considered in service delivery^50^. The involvement of multiple professionals, combined with the stressful and chaotic nature of crisis presentation, often results in confusion about the responsible clinician or team, and the treatment plan^36^. This impedes motivation to engage in mental health aftercare. Repeating the same stories to multiple clinicians may also be another source of frustration^51^. Young people may be less inclined to attend the follow-up appointment if they expect themselves to be repeating the difficult and negative experiences that resulted in their A&E presentation.

Apart from the potential benefits of clinician continuity, there are alternative explanations to the current findings. The patient’s clinical needs might be the determining factor of who offers the follow-up appointment. A different follow-up clinician to the one who initially saw the young person at the hospital might imply step-down care due to a reduction in risks, or might be a part of a specialist service pathway (e.g. neuro-developmental services) in cases where a certain diagnosis is the predominant presentation. Symptom improvement^52^ and clinical diagnosis^53^ are both likely factors of non-attendance. Our findings are thus potentially attributed to the fundamental differences in patients’ clinical profiles instead of the identity of the clinician or team. The non-attendance might also be a reflection of practical limitations. For instance, a discharge that takes place over the weekend or bank holiday may result in a longer time lapse till follow-up, as well as different clinicians who arrange and conduct the appointment due to the shift rota. The former is an established factor of non-attendance^26^ and might have contributed to it in the present study.

The implications of our findings are straightforward. To promote attendance of the first mental health appointment after discharge, services should strive to enable the allocation of the same clinician to see young people in crisis in both hospital and community settings. As soon as the discharge destination is planned, the clinician responsible for the follow-up appointment should seek an opportunity to visit the young person at the hospital, especially for those with a history of non-engagement. It may be less confusing and/or anxiety-provoking if the young person is aware of the arrangement of the follow-up appointment with sufficient notice, understands the rationale and importance of it, and familiarises with whom they will meet. Since this approach likely requires reconfiguration of existing models of community teams, in some regions it may be more sensible to commission and develop bespoke crisis response teams who will be fully responsible for both psychosocial assessment at A&E and the first follow-up appointment in the community. In current practice, the presence of self-harm, or the general level of risks, is commonly a consideration in the allocation of mental health teams in follow-up care. Our findings suggest that early service attendance is not expected to be different between young people who self-harm and those who do not. The same engagement mechanism can and should be utilised to improve follow-up appointment attendance.

This study is subject to at least four limitations. First, data quality might have introduced inaccuracy in attendance. Poor booking practice and incompletion of clinical records are common barriers in service evaluation studies^54^. Although our missing data analyses did not reveal any significant difference between complete and incomplete records, missing data increased the risk of information bias. Second, routine clinical data could not capture the influence of the patient’s parents, which is a significant factor in young people’s access to care and recovery. Further research is needed to explore the specific role and profile of parents of young people who attended follow-up appointments. Third, the medium (face-to-face or remote) of appointments was not recorded for unattended appointments, thus unavailable for analysis. Mixed effects of consultation medium were observed in recent studies^55–58^. Finally, the findings may be region-specific. Although the study’s catchment area covered a large, ethically diverse population, it is unclear whether the results can be generalised across countries or cultures.

In conclusion, children and young people who presented at A&E with mental health crises were three times as likely to attend post-discharge follow-up appointments if they had seen the designated clinician before discharge. Continuity of clinicians should be considered in future mental health policies and service models, to ensure safe community transition for young people in crisis.

### Supplementary information

Supplementary materials

## Data Availability

Electronic health records are, by definition, considered sensitive data in the UK by the GDPR and cannot be shared via public deposition because of information governance restrictions in place to protect patient confidentiality.
