# Supplementary material for: Association between continuity of care and attendance of post-discharge follow-up after psychiatric emergency presentation
Source: Npj Ment Health Res. 2024 Feb 6;3:5. doi: 10.1038/s44184-023-00052-9 (PMC10955912; doi:10.1038/s44184-023-00052-9)
Supplement: Supplementary file 1 — Supplementary materials [file 44184_2023_52_MOESM1_ESM.docx]

**Supplementary materials**

**Missing Data Analyses**

860 records out of 3134 (27%) were determined to be complete. Supplementary Table 1 summarises the characteristics of appointments for complete and incomplete records.

### **Supplementary Table 1.** Summary of characteristics for appointments with complete records (n=860) vs incomplete records (n=2274)

| **Characteristic** | **Not complete (N=2274)** | **Complete (N=860)** |
| --- | --- | --- |
| Borough |  |  |
| Bedfordshire (BD) | 643 (28%) | 32 (4%) |
| City of Hackney (CH) | 342 (15%) | 157 (18%) |
| Luton (LT) | 607 (27%) | 137 (16%) |
| Newham (NH) | 369 (16%) | 267 (31%) |
| Tower Hamlets (TH) | 313 (14%) | 267 (31%) |
| Presentation/thoughts/acts of self-harm at referral |  |  |
| No | 290 (31%) | 202 (23%) |
| Yes | 636 (69%) | 658 (77%) |
| Age at referral (years) | 15 (14-16) | 15 (14-16) |
| Ethnicity |  |  |
| Other | 1286 (57%) | 661 (77%) |
| White British | 986 (43%) | 199 (23%) |
| Looking after child |  |  |
| No | 2149 (95%) | 822 (96%) |
| Yes | 124 (5%) | 38 (4%) |
| Deprivation decile |  |  |
| 1 | 207 (9%) | 95 (11%) |
| 2 | 437 (19%) | 234 (27%) |
| 3 | 354 (16%) | 181 (21%) |
| 4 | 319 (14%) | 163 (19%) |
| 5 | 180 (8%) | 70 (8%) |
| 6 | 175 (8%) | 37 (4%) |
| 7 | 136 (6%) | 15 (2%) |
| 8 | 144 (6%) | 26 (3%) |
| 9 | 163 (7%) | 24 (3%) |
| 10 | 132 (6%) | 15 (2%) |
| Days from referral to follow-up | 2.7 (1.4-6.6) | 5.4 (2.5-7.5) |
| Final follow-up Appointment Medium |  |  |
| Face-to-face | 596 (66%) | 597 (69%) |
| Video | 22 (2%) | 52 (6%) |
| Telephone | 282 (31%) | 211 (25%) |
| Final follow-up Attendance |  |  |
| Attended | 901 (77%) | 860 (100%) |
| Did not attend | 139 (12%) | 0 (0%) |
| Cancelled by client | 32 (3%) | 0 (0%) |
| Cancelled by provider/admitted to hospital | 91 (8%) | 0 (0%) |
| crisis or local team? |  |  |
| Non-crisis (local) team | 365 (31%) | 589 (68%) |
| crisis team | 803 (69%) | 271 (32%) |
| Same team at follow-up |  |  |
| Not same | 383 (33%) | 594 (69%) |
| Same | 785 (67%) | 266 (31%) |
| Same clinician at follow-up |  |  |
| Not same | 918 (79%) | 763 (89%) |
| Same | 250 (21%) | 97 (11%) |

Supplementary Table 2 below describes the odds of having a complete record. Follow-up appointment attendance (primary outcome of interest) was omitted because all attended appointments also had complete records. Referrals from Hackney were slightly more likely to have complete records compared to referrals from other boroughs. Appointments with patients who reported self-harm were more likely to have complete records than appointments with patients who had not reported self-harm. Appointments with White-British patients or patients living in less deprived areas were less likely to have complete records compared to appointments with patients from other ethnic backgrounds or those living in more deprived areas. Appointments offered by crisis teams or those offered by the same clinician/team were less likely to have complete records compared to appointments offered by local teams or those offered by the same clinician/team.

### **Supplementary Table 2**. Predictors of complete record in ELFT dataset (N=3134, complete records=860)

| **Characteristic** | **Crude odds ratio**  **(95% confidence interval)** |
| --- | --- |
| Borough  Hackney  Other | 1.26 (1.03 – 1.55)  1 (base) |
| Age at referral (for every 1-year increase) | 1.03 (0.99 – 1.07) |
| Final follow-up medium  Remote  Face-to-face | 1 (base)  1.16 (0.95 – 1.41) |
| Same clinician at follow-up  Not the same  Same | 1 (base)  0.47 (0.36 – 0.60) |
| Same team at follow-up  Not the same  Same | 1 (base)  0.22 (0.18 – 0.26) |
| Local vs crisis team  Local  crisis | 1 (base)  0.21 (0.17 – 0.25) |
| Self-harm  No  Yes | 1 (base)  1.49 (1.20 – 1.83) |
| Ethnicity  Other  White British | 1 (base)  0.39 (0.33 – 0.47) |
| Looking after child  No  Yes | 1 (base)  0.80 (0.55 – 1.16) |
| Deprivation (for each increase in decile) | 0.84 (0.81 – 0.87) |

Supplementary Table 3 below summarises referral characteristics for those with and without follow-up appointment attendance data specifically. If referrals were missing the follow-up appointment attendance variable, the final follow-up appointment medium variable was also missing, and data on crisis/local and whether or not it was the same team or clinician was sparse.

### **Supplementary Table 3.** Summary of referral characteristics by absence/presence of follow-up appointment attendance data

| **Characteristic** | **No follow-up attendance data (N=1111)** | **Has follow-up attendance data (N=2023)** |
| --- | --- | --- |
| Borough |  |  |
| Bedfordshire (BD) | 87 (8%) | 588 (29%) |
| City of Hackney (CH) | 291 (26%) | 208 (10%) |
| Luton (LT) | 244 (22%) | 500 (25%) |
| Newham (NH) | 267 (24%) | 369 (18%) |
| Tower Hamlets (TH) | 222 (20%) | 358 (18%) |
| Presentation/thoughts/acts of self-harm at referral |  |  |
| No | 246 (33%) | 246 (24%) |
| Yes | 510 (67%) | 784 (76%) |
| Age at referral (years) | 15 (14-16) | 15 (14-16) |
| Ethnicity |  |  |
| Other | 790 (71%) | 1157 (57%) |
| White British | 319 (29%) | 866 (43%) |
| Looking after child |  |  |
| No | 1053 (95%) | 1918 (95%) |
| Yes | 57 (5%) | 105 (5%) |
| Deprivation decile |  |  |
| 1 | 126 (11%) | 176 (9%) |
| 2 | 243 (22%) | 428 (21%) |
| 3 | 211 (19%) | 324 (16%) |
| 4 | 181 (16%) | 301 (15%) |
| 5 | 91 (8%) | 159 (8%) |
| 6 | 66 (6%) | 146 (7%) |
| 7 | 44 (4%) | 107 (5%) |
| 8 | 42 (4%) | 128 (6%) |
| 9 | 51 (5%) | 136 (7%) |
| 10 | 51 (5%) | 96 (5%) |
| Days from referral to follow-up | 24.0 (17.0-38.4) | 3.6 (1.5-6.7) |
| Final follow-up Appointment Medium |  |  |
| Face-to-face | n/a | 1193 (68%) |
| Video | n/a | 74 (4%) |
| Telephone | n/a | 493 (28%) |
| crisis or local team? |  |  |
| Non-crisis (local) team | 3 (60%) | 951 (47%) |
| crisis team | 2 (40%) | 1072 (53%) |
| Same team at follow-up |  |  |
| Not same | 3 (60%) | 974 (48%) |
| Same | 2 (40%) | 1049 (52%) |
| Same clinician at follow-up |  |  |
| Not same | 5 (100%) | 1676 (83%) |
| Same | 0 (0%) | 347 (17%) |

Supplementary Table 4 below summarises the predictors of having follow-up attendance data. Referrals from Hackney were less likely to have follow-up attendance data compared to referrals from other boroughs. Referrals were more likely to have follow-up attendance data if they had reported self-harm or if the patient was White-British or from a higher deprivation decile. The odds ratio (OR) could not be calculated for final follow-up medium or same clinician at follow-up because there was no referral in 1 or more subgroups.

### **Supplementary Table 4**. Predictors of complete record of follow-up appointment attendance data (n=2023)

| **Characteristic** | **Crude odds ratio**  **(95% confidence interval)** |
| --- | --- |
| Borough  Hackney  Other | 0.32 [0.27-0.39]  1 (base) |
| Age at referral (for every 1-year increase) | 1.04 [1.00-1.08] |
| Final follow-up medium | n/a |
| Same clinician at follow-up | n/a |
| Same team at follow-up  Not the same  Same | 1 (base)  1.62 [0.27-9.69] |
| Local vs crisis team  Local  crisis | 1 (base)  1.69 [0.28-10.14] |
| Self-harm  No  Yes | 1 (base)  1.54 [1.25-1.89] |
| Ethnicity  Other  White British | 1 (base)  1.85 [1.58-2.17] |
| Looking after child  No  Yes | 1 (base)  1.01 [0.73-1.41] |
| Deprivation (for each increase in decile) | 1.06 [1.03-1.10] |
